# Circular RNA circSATB2 promotes progression of non-small cell lung cancer cells

**DOI:** 10.1186/s12943-020-01221-6

**Published:** 2020-06-03

**Authors:** Nan Zhang, Aruo Nan, Lijian Chen, Xin Li, Yangyang Jia, Miaoyun Qiu, Xin Dai, Hanyu Zhou, Jialu Zhu, Han Zhang, Yiguo Jiang

**Affiliations:** 1 State Key Laboratory of Respiratory Disease, The First Affiliated Hospital of Guangzhou Medical University, Xinzao, Panyu District, Guangzhou, 511436 People’s Republic of China; 2 Institute for Chemical Carcinogenesis, Guangzhou Medical University, Xinzao, Panyu District, Guangzhou, 511436 People’s Republic of China

**Keywords:** Lung cancer, Progression, circRNA, Exosome, miRNA

## Abstract

**Background:**

Lung cancer has high morbidity and mortality worldwide with non-small cell lung cancer (NSCLC) accounting for 85% of the cases. Therapies for lung cancer have relatively poor outcomes and further improvements are required. Circular RNAs have been reported to participate in the occurrence and progression of cancer. Information on the functions and mechanism of circRNAs in lung cancer is limited and needs more exploration.

**Methods:**

We detected expression of genes and proteins by qPCR and western blot. Function of circSATB2 was investigated using RNA interference and overexpression assays. Location of circSATB2 was assessed by fluorescence in situ hybridization (FISH). Interaction of circSATB2, miR-326 and *FSCN1* was confirmed by dual-luciferase reporter assay.

**Results:**

Data from the investigation showed that circSATB2 was highly expressed in NSCLC cells and tissues. circSATB2 positively regulated fascin homolog 1, actin-bundling protein 1 (FSCN1) expression via miR-326 in lung cancer cells. Furthermore, circSATB2 can be transferred by exosomes and promote the proliferation, migration and invasion of NSCLC cells, as well as induce abnormal proliferation in normal human bronchial epithelial cells. Also, circSATB2 was highly expressed in serumal exosomes from lung cancer patients with high sensitivity and specificity for clinical detection and was related to lung cancer metastasis.

**Conclusions:**

circSATB2 participated in the progression of NSCLC and was differentially expressed in lung cancer tissue and serumal exosomes. circSATB2 may be potential biomarker for the diagnosis of NSCLC.

## Background

Lung cancer is one of the most common malignant tumors worldwide, with serious impacts on human health [1]. Lung cancer has been the leading cause of cancer-related deaths worldwide, with non-small cell lung cancer (NSCLC) accounting for 85% of lung cancer cases [2, 3]. Despite extensive research and progress, therapies for lung cancer remain inadequate and further improvements are needed [4]. There is thus an urgent need to further our scientific understanding of lung cancer and to identify early diagnostic biomarkers and new therapeutic targets.

Exosomes are extracellular membranous microvesicles with a diameter of 40–160 nm secreted by various cell types. Exosomes derived from host cells may be taken up by adjacent or distant cells and exert their biological roles in these receptor cells [5]. Exosomes have been implicated in the occurrence and development of many diseases, including cancer [6–8]. Exosomes include biological molecules such as lipids, proteins, DNA and non-coding RNAs (ncRNAs). Exosomes secreted by different host cells contain different molecules and therefore have different biological functions. Exosomes containing functional ncRNAs can contribute to the progression of diseases, including cancer [9, 10].

ncRNAs are a class of RNAs that do not code for proteins and comprise approximately 98% of the human genome [11]. ncRNAs have become an important topic in the field of epigenetics, and new types and functions of ncRNAs are being explored. Most studies of ncRNAs have focused on microRNAs (miRNAs), long ncRNAs (lncRNAs) and circular RNAs (circRNAs). circRNAs have attracted the attention of researchers because of their special structure and important functions [12, 13]. circRNAs are abundant in the human body and have multiple biological functions [14], including acting as “sponges” for miRNAs, thus regulating gene expression at the post-transcriptional level [15]. Previous studies have indicated that circRNAs are closely related to human cancers [16–19]. However, the roles of circRNAs in the occurrence and development of lung cancer need to be further explored.

circRNAs can be packaged into exosomes and transferred to receptor cells, further impacting the development of diseases [20]. However, information on the functions and mechanism of circRNAs in NSCLC is limited. In the current study, we investigated the function and molecular mechanism of circSATB2 in the proliferation, migration and invasion of NSCLC, and explored its potential as a biomarker of NSCLC and lung cancer metastasis. Our results suggest that circSATB2 could regulate fascin homolog 1, actin-bundling protein 1 (FSCN1) expression via direct binding to miR-326, further impacting the progression of lung cancer. In addition, circSATB2 participated in cell-cell communication via exosomes. Results of this study revealed a novel mechanism of lung cancer progression and suggested a potential new molecular target for lung cancer research.

## Method

### Cell culture

Immortalized human bronchial epithelial cells (BEAS-2B) and human lung cancer cell lines (A549, H460, H1299, H226, MES-1) were purchased from Guangzhou Saiku Biotechnology Co. (Guangzhou, China), and human embryonic kidney cells (HEK-293 T) were purchased from the Cell Bank of Shanghai Academy of Chinese Sciences. BEAS-2B cells were cultured in BEBM serum-free medium (CC-3170, Lonza, Verviers, Belgium), A549, H460, H226, MES-1 and H1299 cells were cultured in RPMI-1640 medium (Hyclone, Logan, Australia), and HEK-293 T cells were cultured in DMEM (Hyclone) containing 10% fetal bovine serum (Every Green, Zhejiang, China) at 37 °C and 5% CO_2_ under saturated humidity. Exosomes in fetal bovine serum were removed by ultracentrifugation before the experiment.

### Lung cancer tissues and serum

Fifty-nine paired NSCLC and matched normal adjacent tissue samples were obtained from Guangzhou General Hospital of Guangzhou Military Command (Guangzhou, China). Surgically resected tissues were frozen in liquid nitrogen. The tissue and serum samples were not obtained from the same set of individuals. Serum samples of lung cancer patients and people not diagnosed with lung cancer were obtained from the Cancer Center of Guangzhou Medical University and frozen at − 80 °C. This study Exosomes were isolated from cell culture medium supernatant by ultracentrifugation. Cell culture medium was collected and centrifuged at 300×*g* for 10 min, 2000×*g* for 10 min and 10,000×*g* for 30 min to remove residual live cells, dead cells and cell debris, respectively. The supernatant was then collected and centrifuged at 120,000×*g* for 90 min at 4 °C to precipitate the exosomes. Exosome precipitates were washed with phosphate-buffered saline (PBS) for purity and then resuspended in PBS for further research. Serum from lung cancer and non-cancerous donors was centrifuged at 2000×*g* for 30 min at 4 °C, the supernatant was collected and the exosomes were isolated using Total Exosome Isolation Reagent (Invitrogen, Carlsbad, CA, USA) according to the manufacturer’s protocol. For morphology observation, the copper electron microscopy grids were floated above the exosome suspension for 3 min, then floated above phosphotungstic acid for 3 min. The grids were dried and the morphology and size of the exosomes were observed by transmission electron microscopy (TEM) using the Tecnai G2 Spirit (FEI, Hillsboro, OR, USA). The concentration, size distribution and zeta potential of isolated exosomes were detected by nanoparticle tracking analysis using a NanoSight NS300 (Malvern, UK). TSG101, CD9 and CD63 proteins were used as markers to identify exosomes by western blotting.

### Total RNA extraction and quantitative reverse transcription (qRT)-PCR

Total RNA was extracted from cells using Trizol reagent (Invitrogen) and total RNA was isolated from exosomes using an Exosomal RNA and Protein Extraction Kit (101Bio, Mountain View, CA, USA), according to the manufacturers’ protocols. The quality and concentration of the purified total RNAs were detected using a NanoDrop1000 spectrophotometer (NanoDrop Technologies, Wilmington, DE, USA) and then reverse-transcribed using the Goscript™ Reverse Transcription System (Promega, Madison, WI, USA). The RNA sample was divided into two uniform parts before circRNA reverse transcription. One part was treated with RNase R (Epicentre, Madison, WI, USA) for 10 min at 37 °C for the further detection of circRNA. The other part was treated with RNase R-free water for the final detection of GAPDH and other linear genes. qRT-PCR was carried out using GoTaq® qPCR Master Mix (Promega) according to the manufacturer’s procedure, using an Applied Biosystems 7500 Real-Time PCR System (Applied Biosystems, Foster City, CA, USA). GAPDH and U6 were used as internal references and cel-miR-39 as an external reference. Relative expression levels of exosomal circSATB2 in human serum were calculated using the 2^-ΔCt^ method, and all other PCR reactions were calculated using the 2^−ΔΔCt^ method.

### Establishment of circSATB2 stably transfected cell lines

Stable lentivirus-3 circSATB2-shRNA and lentivirus-5 circSATB2-OE vectors were constructed and the lentiviruses were packaged and purified by Shanghai GenePharma Co., Ltd. (Suzhou, China). Lentiviral vectors including lv5 (control for the OE group), circSATB2-OE (circSATB2 overexpression), lv3 (control for the knockdown group) and circSATB2-sh (circSATB2 knockdown) were used to infect the cells according to the manufacturer’s protocol. Cells were incubated for more than 48 h and fluorescent signals were observed using a fluorescence microscope (AMG EVOS, Mill Creek, WA, USA). Puromycin was used for selecting stable strains.

### EdU assay

Cell proliferation was detected using the EdU assay. 1 × 10^4^ A549, H460 and H1299 cells stably transfected with circSATB2 were seeded on 96-well plates and cultured in medium containing 10% fetal bovine serum at 37 °C and 5% CO_2_ for 48 h. 1 × 10^4^ A549, H460 and H1299 cells were seeded on 96-well plates and cultured with 10% exosome-free fetal bovine serum for 24 h. Exosomes expressing high or low levels of circSATB2 (about 3 × 10^7^ exosomes per 10,000 cells) were then added to the plates and co-cultured with the A549, H460 and H1299 cells for 48 h; cells in the control group were co-cultured with the same volume of PBS. EdU assays were then performed according to manufacturer’s protocol using the Cell-Light EdU Apollo 567 in vitro imaging kit (RiboBio, Guangzhou, China). Images were captured using a fluorescence microscope (AMG EVOS).

### Wound healing assay

About 5 × 10^5^ A549, H460 and H1299 cells stably transfected with circSATB2 were cultured in medium containing 1% fetal bovine serum on 6-well plates. As a control, the same number of A549, H460 and H1299 cells were cultured with 1% exosome-free fetal bovine serum. When the cells reached 100% confluence, streaks were created across the monolayer using a 200 μL pipette tip. Cells were washed twice with PBS and the circSATB2-transfected cells and cells co-cultured with exosomes were cultured with serum-free RPMI-1640 medium at 37 °C and 5% CO_2_ for 24 h to reduce the influence of cell proliferation. Images of the cells were captured at 0 and 24 h after wounding using a microscope (TS100-F, Nikon, Tokyo, Japan).

### Transwell migration and invasion assay

Migration assay was performed using Transwell pore polycarbonate membrane insert (Corning, New York, USA), invasion assay was performed using Matrigel-coated invasion chambers (BD Biocoat, Corning). Cells were seeded into the upper chamber coated with matrigel, with or without exosome treatment, and cultured in serum-free medium. The lower chamber contained medium supplemented with 20% normal or exosome-free fetal bovine serum. Cells invading the membrane and adhering to the lower surface after 24 h were fixed with methanol and then stained with crystal violet for 20 min. Cells adhering to the lower surface were then washed with PBS and cells on the upper membrane were removed. Images of the stained cells were captured using a microscope (Nikon).

### Cell viability assay

Cells were seeded in 96-well plates and were cultured for 24 h, treated with exosomes and co-cultured for 48 h. Then 10 μL of CCK-8 solution (Dojindo, Mashikimachi, Japan) was added to each well and incubated for 1 h at 37 °C. The absorbance at 450 nm was then measured.

### Colon formation assay

BEAS-2B cells were plated on a 6-well plate. Cells were cultured at 37 °C and 5% CO_2_ for 10–14 days with circSATB2-sh and circSATB2-OE exosomes treatment. Cells were fixed with methanol and then stained with crystal violet for 20 min, then observed the colony formation colony formation.

### Exosome uptake assay

For intracellular staining, SYTO®RNA Select™ green fluorescent cell stain (Life Technologies, Carlsbad, CA, USA) was diluted with DMSO at 37 °C for nucleic acid staining. Cells were co-cultured with the staining solution for 2 h and exosomes were then isolated from the culture medium supernatant. Purified exosomes were added to lung cancer cells and incubated for 6 h, the cells were then washed with PBS, and the nucleus was stained with DAPI. Images of cells were captured using fluorescence microscopy (AMG EVOS).

For extracellular staining, exosomes were extracted and transfected using Texas Red-labeled siRNA from an Exo-Fect™ Exosome Transfection Kit (SBI, Palo Alto, CA, USA) according to the manufacturer’s protocol. Transfected exosomes were added to lung cancer cells and incubated for 6 h. Images of the cells were captured using fluorescence microscopy (AMG EVOS).

### Dual luciferase reporter assay

Fluorescein-labeled reporter gene detection was carried out using a Dual Luciferase Assay System kit (Promega) after sequence comparisons of circSATB2, *FSCN1* and miRNAs, according to the manufacturer’s instructions. Wild-type and mutant pmiRGlo-circSATB2 and pmiRGlo-*FSCN1*–3′UTR dual luciferase reporter vectors incorporating miRNA binding sites were constructed by Guangzhou Yingxin Co., Ltd. (Guangzhou, China). HEK-293 T cells were seeded on 12-well plates and cultured for 24 h, and then co-transfected with wild-type or mutant vectors with miRNA mimics using transfection reagents. After 48 h incubation, passive lysis buffer was added to each well and the supernatant was collected for detection. Passive lysis buffer sample (20 μL) and 100 μL of Luciferase Assay Reagent II were added to each well of 96-well plates and the absorbance was measured at 580 nm using a microplate reader. Stop & Glo® Reagent (100 μL) was then added to the same well and the absorbance was measured at 460 nm.

### Western blot

Proteins were extracted from exosomes using an Exosomal RNA and Protein Extraction Kit (101Bio), according to the manufacturer’s protocol. Total proteins were extracted by RIPA Lysis Buffer (Beyotime, Shanghai, China) and quantified using a Pierce BCA Protein Assay Kit (Thermo Fisher Scientific, Waltham, MA, USA). Proteins (25 μg) were loaded and separated on 10% SDS-PAGE gels, concentrated on 5% SDS-PAGE gels, and then transferred to polyvinylidene difluoride membranes (Merck Millipore, Billerica, MA, USA). After 2 h blocking with 5% bovine serum albumin, the membranes were incubated with primary anti-GAPDH, anti-TSG 101, anti-CD9, anti-CD63 and anti-FSCN1 antibodies (Abcam, Cambridge, UK) overnight at 4 °C, dilutions of all antibodies were performed as recommended in the instructions. The membranes were then incubated with goat anti-rabbit IRDye™ 800CW or goat anti-mouse IRDye™ 800CW secondary antibody at room temperature for 1 h. Finally, images were scanned and analyzed using an Odyssey Imaging System (LI-COR, Lincoln, NE, USA).

### Transfection experiment

The miRNA mimic and inhibitor were transfected using a RiboFECT™ CP Transfection Kit (RiboBio), and plasmids were transfected using EndoFectin-Lenti (GeneCopoeia, Rockville, MD, USA) reagent, according to the manufacturers’ protocols.

### Fluorescence in situ hybridization (FISH)

A specific circSATB2 FISH probe labeled with 6-FAM was designed and used in the experiment. Cells attached to slides were immobilized with 4% paraformaldehyde, washed with PBS, and then digested by protease K (Sangon, Shanghai, China) at 37 °C for 5 min. After washing with PBS, the cells were immobilized with 1% paraformaldehyde followed by successive dehydration in 70, 85, and 100% alcohol. Hybridization solution dilute probe was dripped onto the cell slide followed by denaturation at 73 °C for 3 min and hybridization overnight at 42 °C in the dark. The slides were then washed with 50% formamide/2 × SSC preheated to 43 °C, 0.1% NP-40/2 × SSC preheated to 37 °C, and DAPI staining solution at room temperature. Images were acquired using a laser confocal microscope (Leica, Mannheim, Germany).

### Cytoplasm and nuclear localization

The cytoplasm and nuclear fractions were separated and isolated from cells using a PARIS kit (Ambion, Austin, TX, USA) according to the manufacturer’s protocol. Cytoplasmic and nuclear RNAs were then converted to cDNAs and detected and analyzed by qPCR.

### Statistical analysis

All experiments in this study were performed independently with at least three biological replicates. Data are presented as means±standard deviation. Statistical analyses were performed using GraphPad Prism7 (GraphPad, San Diego, CA, USA) and SPSS 22.0 software (IBM, Armonk, NY, USA). Differences between two groups were analyzed by independent sample *t*-tests and differences among multiple groups by one-way ANOVA. Clinical characteristics were analyzed by χ^2^ tests. Correlations were analyzed by Pearson correlation test. Receiver operating characteristic (ROC) curves were prepared and the area under the ROC curve (AUC) was determined to analyze the sensitivity and specificity of circSATB2 detection. A value of *P* < 0.05 indicated a statistically significant difference.

## Results

### Highly expressed circSATB2 promotes proliferation, migration and invasion of NLCSC cells

**Fig. 1:**
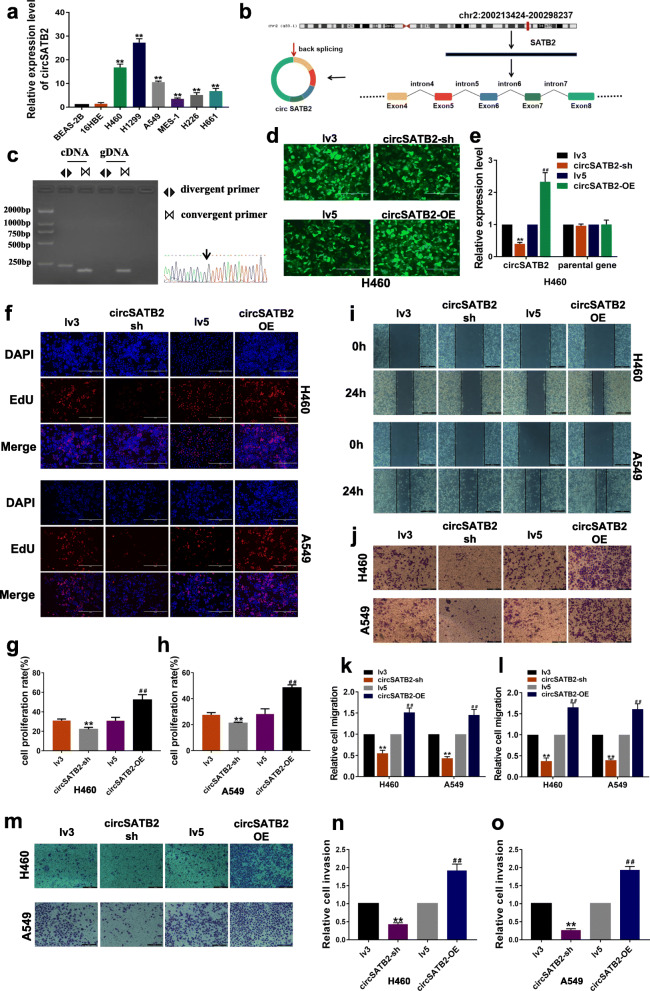
circSATB2 promotes proliferation, migration and invasion of NCSLC cells. **a** circSATB2 expression in normal and lung cancer cells. **b** Genomic location and splicing mode of circSATB2. **c** Identification of circSATB2 qPCR amplification products. DNA gel electrophoresis (left) and Sanger sequencing of qPCR product (right). **d** GFP-tagged lentivirus in cells observed by fluorescence microscope. **e** Efficiency of stably transfected circSATB2-knockdown and overexpressing H460 cells detected by qPCR. **f** EdU assay to detect proliferation of circSATB2-knockdown and overexpressing stably transfected H460 and A549 cells. **g**, **h** Quantitative analysis of EdU assay. **i** Wound healing assay to detect migration of circSATB-knockdown and overexpressing stably transfected H460 and A549 cells. **j** Transwell migration assay detect migration of circSATB-knockdown and overexpressing stably transfected H460 and A549 cells. **k** Quantitative analysis of wound healing assay. (l) Quantitative analysis of Transwell migration assay. **m** Transwell invasion assay to detect invasion of circSATB2 knockdown and overexpressing stably transfected H460 and A549 cells. **n**, **o** Quantitative analysis of Transwell invasion assay. All experiments were repeated independently three times. Data are presented as means±standard deviation. ^**^P < 0.01 compared with BAES-2B cells or lv3 group; ^##^*P* < 0.01 compared with lv5 group

We selected seven circRNAs (circ0005542, circ0070440, circ0008012, circ0000592, circ0008928, circ0004771 and circ0001095) that were recorded as highly expressed in cancer exosomes by the exoRBase database (http://www.exorbase.org). To determine if these differentially expressed exosomal circRNAs were functional in lung cancer, we assessed their expression in normal human bronchial epithelial cells (BEAS-2B and 16HBE cells) and NLCSC cells (H1299, H226, MES-1, H460, H661 and A549 cells). circ0008928 (named circSATB2) was highly and stably expressed in H460, A549 and H1299 cells compared with BEAS-2B cells (Fig. 1a, Additional file 1: Figure S1A), indicating a potential role in NSCLC. circSATB2 consists of exon 4 to exon 8 of SATB2 and is located on chromosome 2q33.1 (Fig. 1b). We also performed qPCR using circSATB2 divergent primers containing a back-splice junction and convergent primers for both cDNA and gDNA (sequence of primers were shown in Additional file 2: Table S1) and discovered that divergent primers only amplified a band in cDNA (Fig. 1c), while circSATB2 also showed resistance to RNase R (Additional file 1: Figure S1B). To investigate the function of circSATB2 in A549, H460 and H1299 cells, we established stable circSATB2 knockdown (circSATB2-sh) and overexpression (circSATB2-OE) cell lines, as well as their respective negative controls lv3 and lv5. Fluorescence signals were observed using a fluorescence microscope to verify that cells were successfully transfected with lentivirus, and circSATB2 knockdown and overexpression efficiency were analyzed by qPCR. Expression of circSATB2 and not the parental gene was changed (Fig. 1d, e; Additional file 1: Figure S1C, D). Cell proliferation was enhanced by overexpression and impaired by knockdown of circSATB2 in A549, H460 and H1299 cells, as detected by EdU assay (Fig. 1f-h; Additional file 1: Figure S1E, F), compared with the lv3 and lv5 control groups, respectively. We also investigated the roles of circSATB2 in migration and invasion of A549, H460 and H1299 cells using wound healing, Transwell migration and invasion assays. circSATB2 knockdown significantly decreased the migration of H460, A549 and H1299 cells compared with the lv3 group, and the opposite result was observed in the circSATB2-OE cells compared with the lv5 group (Fig. 1i-l, Additional file 1: Figure S1G-J). The invasive capacity of H460, A549 and H1299 cells increased in the circSATB2-OE group but decreased in the circSATB2-sh group compared with the lv5 and lv3 groups, respectively (Fig. 1m-o; Additional file 1: Figure S1K, L). Collectively, these results demonstrate that circSATB2 was highly expressed in H460, A549 and H1299 cells and significantly affected their proliferation, migration and invasion.

### circSATB2 directly binds miR-326 and regulates miR-326 expression in NSCLC cells

**Fig. 2:**
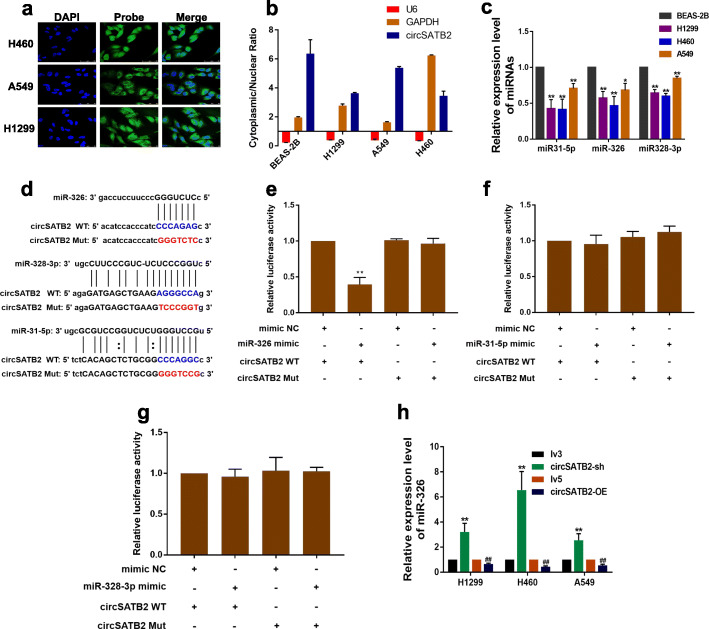
circSATB2 directly binds to miR-326 and regulates miR-326 expression in NSCLC cells. **a** FISH for subcellular localization of circSATB2. 6-FAM labeled the circSATB2 FISH probe, DAPI stained cell nuclei. **b** Cytoplasmic and nuclear circSATB2 expression detected by qPCR. U6 and GAPDH acted as nuclear and cytoplasmic reference genes, respectively. Relative expression measured by the 2^-(cytoplasmic Ct value - nuclear Ct value)^ method. **c** miRNA expression in BEAS-2B and NSCLC cells detected by qPCR. **d** Sequence alignments between circSATB2 and seed sequences of miRNAs. WT, wild-type vector; Mut, mutant sequence vector of circSATB2. **e**–**g** Dual luciferase reporter gene assay to detect interaction between circSATB2 and miRNAs. Mimic NC, empty vector control; mimic, miRNA mimic. **h** miR-326 expression in circSATB2-sh and circSATB2-OE stably transfected lung cancer cells detected by qPCR. All experiments repeated independently three times. Data are presented as means±standard deviation. ^*^P < 0.05, ^**^P < 0.01 compared with BEAS-2B cells (3**c**), mimic NC group (3**e**-**g**), and lv3 group (3 **h**); ^##^*P* < 0.01 compared with lv5 group

To further explore the molecular mechanism of circSATB2 in regulating the proliferation, migration, and invasion of NSCLC cells, we analyzed its capacity for protein-coding via ORF finder (http://www.ncbi.nlm.nih.gov/orffinder/); the results indicated circSATB2 lacked the ability to encode protein. We then designed a specific circSATB2 probe labeled with 6-FAM (shown in Additional file 2: Table S2) to detect its intracellular distribution by in situ hybridization. The results showed that circSATB2 was mainly distributed in cytoplasm (Fig. 2a). We also separated the cytoplasm from the nucleus and detected the subcellular location of circSATB2 by qPCR. This confirmed that circSATB2 was mainly expressed in the cytoplasm (Fig. 2b), consistent with the previous result from in situ hybridization.

The above results suggested that circSATB2 may function at the post-transcriptional level. Target proteins of circSATB2 recorded in CSCD (http://gb.whu.edu.cn/CSCD/) showed that circSATB2 may interact with AGO2, indicating a relationship between circSATB2 and miRNAs. We obtained miRNAs potentially binding circSATB2 using RegRNA (http://regrna.mbc.nctu.edu.tw/html/prediction.html) and CSCD and found 186 miRNA binding sites. We then chose miR31-5p, miR-326 and miR328-3p, which have been reported to be associated with cancer progression [21–23] for further study. We detected the expression of these three miRNAs in normal BEAS-2B, H460, A549 and H1299 cells by qPCR (sequence of primers were shown in Additional file 2: Table S1), while all three were downregulated in H460, A549 and H1299 cells compared with BEAS-2B cells (Fig. 2c). We further constructed wild-type and mutant pmiRGlo-circSATB2 vectors incorporating miRNA binding sites (Fig. 2d) for dual luciferase reporter assays, and co-transfected these vectors with miRNA mimics to determine if circSATB2 could bind to the three miRNAs. Results showed the circSATB2 directly bound to miR-326 (Fig. 2e), but not to miR-31 or miR-328 (Fig. 2f, g). qPCR showed that miR-326 expression was increased in circSATB2-sh and decreased in circSATB2-OE H460, A549 and H1299 cells, compared with the lv3 and lv5 groups, respectively (Fig. 2h). indicating that circSATB2 could negatively regulate miR-326 expression. These results thus demonstrated that circSATB2 was mainly located in the cytoplasm and acted at the post-transcriptional level by binding directly to miR-326 and negatively regulating miR-326 expression.

### circSATB2 regulates FSCN1 expression via miR-326

**Fig. 3:**
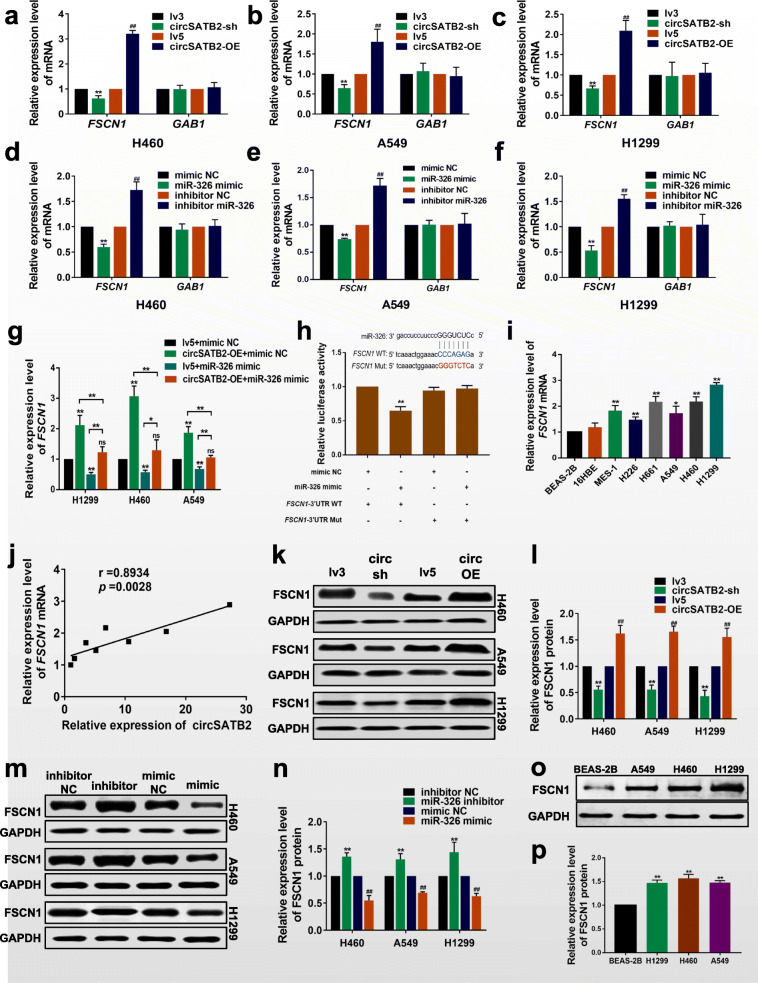
circSATB2 regulates FSCN1 expression via miR-326. **a**–**c***FSCN1* and *GAB1* mRNA expression in circSATB2-knockdown and overexpressing stably transfected lung cancer cells detected by qRT-PCR. **d**–**f***FSCN1* and *GAB1* mRNA expression in miR-326-knockdown and overexpressing lung cancer cells detected by qRT-PCR. **g***FSCN1* mRNA expression in NSCLC cells after co-transfection with circSATB2 and miR-326 overexpression vectors. **h** Sequence alignments (upper) and interaction between miR-326 and FSCN1 detected by dual luciferase reporter gene assay (under). WT, wild-type vector; Mut, mutant sequence vector of *FSCN1*; mimic NC, empty vector control; mimic, miR-326 mimic. **i**, **j** Correlation between circSATB2 and *FSCN1* mRNA expression in NSCLC cells. **k**, **l** FSCN1 protein expression in circSATB2-knockdown and overexpressing stably transfected H460, A549 and H1299 cells detected by western blot. **m**, **n** FSCN1 protein expression in miR-326-knockdown and overexpressing H460, A549 and H1299 cells detected by western blot. **o**, **p** FSCN1 protein expression in BEAS-2B, H460, A549 and H1299 cells detected by western blot. All experiments were independently repeated three times. Data are presented as means±standard deviation. ^**^P < 0.01 compared with BEAS-2B, lv3, or inhibitor NC group; ^#^*P* < 0.05, ^##^*P* < 0.01 compared with lv5 group

We predicted 133 potential target genes of miR-326 using miRBase (https://www.mirbase.org) and TargetScan (http://www.targetscan.org/vert_72/). *FSCN1* and GRB2-associated binding protein 1

 (*GAB1*), which were reported to be involved in the progression of lung cancer [24, 25], were identified as potential targets of miR-326. To determine if circSATB2 or miR-326 could regulate *FSCN1* and *GAB1* expression, we detected *FSCN1* and *GAB1* expression in circSATB2- and miR-326 knockdown and overexpressing NSCLC cells via qPCR (sequence of primers were shown in Additional file 2: Table S1). *FSCN1* expression was significantly decreased by circSATB2 knockdown and increased by circSATB2 overexpression compared with the lv3 and lv5 groups, respectively, but there were no significant alterations in *GAB1* (Fig. 3a-c). *FSCN1* expression was also significantly decreased by miR-326 overexpression and increased by miR-326 knockdown compared with the mimic empty vector control (mimic NC) and inhibitor NC groups, while no significant alterations in *GAB1* were detected (Fig. 3d-f). *FSCN1* was therefore selected as the miR-326 target for further study.

To verify if circSATB2 could regulate *FSCN1* mRNA level via miR-326, we transfected the circSATB2-OE A549, H460 and H1299 cells with miR-326 mimics. Results showed circSATB2 overexpression increased *FSCN1* mRNA levels while miR-326 overexpression decreased *FSCN1* mRNA levels, compared with the lv5 + miR-326 mimic NC group. *FSCN1* mRNA levels in the circSATB2-OE + miR-326 mimic group decreased compared with the circSATB2-OE + miR-326 mimic NC group; indicating that miR-326 overexpression could rescue the increased *FSCN1* mRNA levels induced by circSATB2 (Fig. 3g). These results suggested that *FSCN1* may be positively regulated by circSATB2 via miR-326.

To investigate the interaction between miR-326 and *FSCN1,* wild-type and mutant pmiRGlo-*FSCN1*–3′ untranslated region (UTR) vectors incorporating miR-326 binding sites were constructed for dual luciferase reporter gene detection. Luciferase activity was significantly decreased after co-transfection with the wild-type vector and miR-326 mimics, but not after transfection with the mutant vector (Fig. 3h), indicating that miR-326 could directly bind to *FSCN1* mRNA. Expression of FSCN1 and circSATB2 were positively correlated in NSCLC cells (Fig. 3i and j). We also detected FSCN1 protein expression in NSCLC cells after circSATB2 and miR-326 knockdown or overexpression. circSATB2 knockdown notably decreased FSCN1 protein expression while circSATB2 overexpression increased it, compared with the lv3 and lv5 groups, respectively (Fig. 3k, l). FSCN1 protein expression was also significantly decreased by miR-326 overexpression and increased by miR-326 knockdown, compared with miR-326 mimic NC and miR-326 inhibitor NC, respectively (Fig. 3m, n). These results indicated that FSCN1 protein expression was positively regulated by circSATB2 and negatively regulated by miR-326. Western blot analysis showed that FSCN1 protein levels were also higher in H460, A549 and H1299 cells compared with BEAS-2B cells (Fig. 3o, p).

### Expression of circSATB2, miR-326, and *FSCN1* in NSCLC tissues

**Fig. 4:**
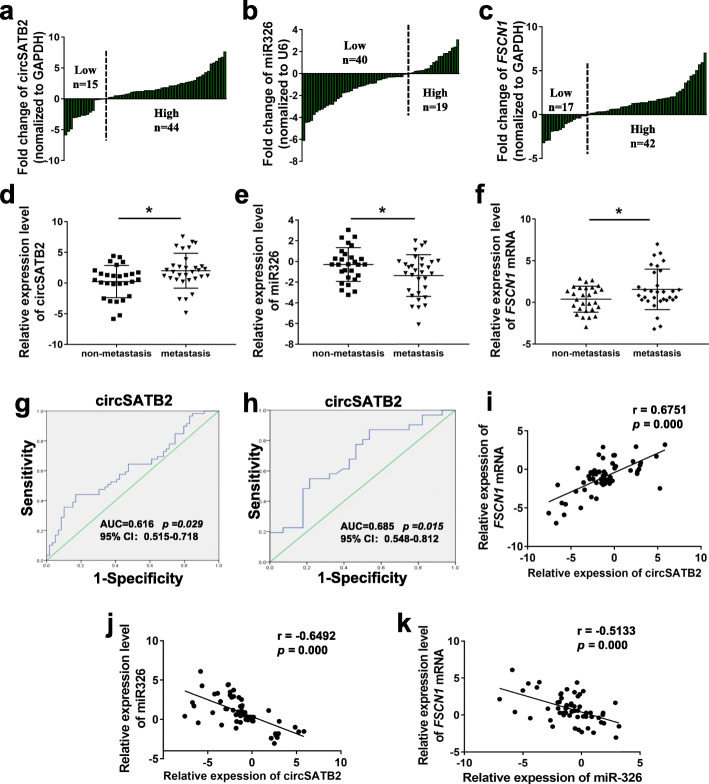
circSATB2, miR-326 and *FSCN1* expression in NSCLC tissues. **a**–**c** Expression of circSATB2, miR-326 and *FSCN1* in lung cancer and paired normal adjacent tissues detected by qPCR. **d**–**f** Expression of circSATB2, miR-326 and *FSCN1* in metastatic and non-metastatic lung cancer tissues detected by qPCR. **g** ROC curve analyses and AUC values for circSATB2 in lung cancer and normal adjacent tissues. **h** ROC curve analyses and AUC values for circSATB2 in metastatic and non-metastatic lung cancer tissues. **i**–**k** Correlation between circSATB2, miR-326 and *FSCN1* expression in NSCLC tissues. Experimental data are presented as means±standard deviation. ^*^P < 0.05, compared with non-metastatic lung cancer tissues

We detected the expression of circSATB2, miR-326, and *FSCN1* in 59 sets of paired NSCLC and normal adjacent tissues by qPCR. circSATB2 and *FSCN1* were highly expressed while miR-326 was weakly expressed in lung cancer compared with normal adjacent tissues (Fig. 4a-c). Analysis of the clinical characteristics of these cohorts suggested that circSATB2, miR-326, and *FSCN1* expression levels were related to lung cancer lymphatic metastasis (Additional file 2: Tables S3-S5). circSATB2 and *FSCN1* were upregulated and miR-326 was downregulated in metastatic compared with non-metastatic lung cancer tissues (Fig. 4d-f).

ROC curves and area under the ROC curves (AUC) were then analyzed to assess the sensitivity and specificity of circSATB2 for the diagnosis of lung cancer and metastatic NSCLC patients, comparing NSCLC and normal adjacent tissues as well as metastatic and non-metastatic lung cancer tissues. In NSCLC tissues, the AUC values were 0.616 for circSATB2 (Fig. 4g). In metastatic NSCLC tissues, the AUC values were 0.685 for circSATB2 (Fig. 4h). Pearson correlation analysis indicated a positive correlation between circSATB2 and *FSCN1* expression, while both circSATB2 and *FSCN1* expression were negatively correlated with miR-326 expression (Fig. 4i-k). These results indicated that circSATB2, miR-326, and *FSCN1* were differentially expressed in NSCLC tissues and metastatic NSCLC tissues, and that circSATB2, miR-326, and *FSCN1* expression were related to lung cancer lymphatic metastasis. The correlations between circSATB2, miR-326 and *FSCN1* expression also support their potential role in NSCLC metastasis and the interaction between circSATB2, miR-326, and *FSCN1*.

### circSATB2 participates in cell-to-cell communication via exosomes

**Fig. 5:**
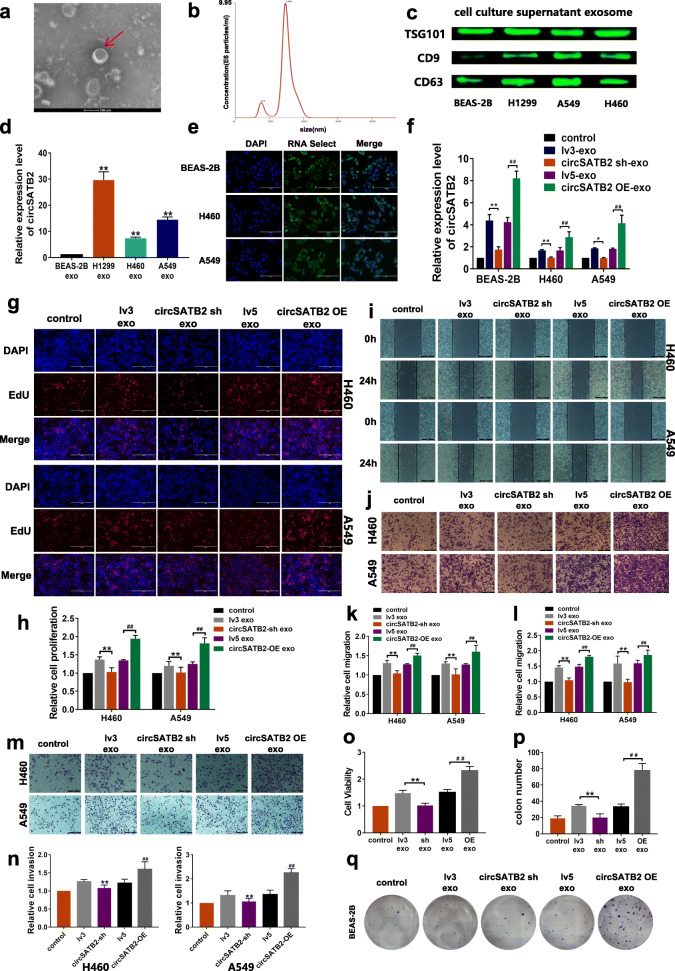
circSATB2 affects cell proliferation, migration and invasion of NSCLC cells and increases proliferation of normal cells via exosomes. **a** Exosome identification by TEM. **b** Nanosight-detected size range of cellular exosome diameters. **c** Western blot detected expression of exosome marker proteins TSG101, CD9 and CD63. **d** circSATB2 expression in exosomes derived from normal bronchial epithelial cells and NSCLC cells detected by qPCR. **e** Exosomes from fluorescence-labeled cells were taken up by BEAS-2B and NSCLC cells. **f** circSATB2 expression after co-culture with circSATB2-OE and circSATB2-sh exosomes detected by qPCR. **g**, **h** EdU assay to detect proliferation of H460 and A549 cells after co-culture with circSATB2-sh and circSATB2-OE exosomes. **i**, **k** Wound healing assay to detect migration of H460 and A549 cells after co-culture with circSATB2-sh and circSATB2-OE exosomes. **j**, **l** Transwell migration assay to detect migration of H460 and A549 cells after co-culture with circSATB2-sh and circSATB2-OE exosomes. **m**, **n** Transwell invasion assay to detect invasion of H460 and A549 cells after co-culture with circSATB2-sh and circSATB2-OE exosomes. **o** CCK-8 assay to detect the cell viability of BEAS-2B cells after co-culture with circSATB2-sh and circSATB2-OE exosomes. **p**, **q** Colon formation assay to detect the proliferation of BEAS-2B cells after co-culture with circSATB2-sh and circSATB2-OE exosomes. All experiments were repeated independently three times. Data are presented as means±standard deviation. ^**^P < 0.01 compared with BEAS-2B-exo or lv3-exo group; ^#^*P* < 0.05, ^##^*P* < 0.01 compared with lv5-exo group

To determine if circSATB2 could be transferred by exosomes and affect the function of recipient cells, we performed experiments to co-culture the BEAS-2B, H460 and A549 cells with exosomes containing higher or lower circSATB2 levels. We extracted exosomes from normal and lung cancer cells via ultracentrifugation and then observed the morphology as well as analyzed the characteristics of the isolated exosomes. The extracted exosomes showed a typical membrane structure and diameter less than 100 nm observed by TEM (Fig. 5a). Nanoparticle tracker analysis (NTA) also showed a concentration of 3.95 × 10^9^ ± 4.23 × 10^8^/mL and zeta potential values of − 17.67 ± 2.092 mV, while the size distribution of the exosomes was within 200 nm, with a mode of 147.7 nm (Fig. 5b). Expression of the exosome marker proteins TSG101, CD9 and CD63 was detected by western blot (Fig. 5c). We then detected circSATB2 expression in exosomes derived from BEAS-2B, H460, A549 and H1299 cells and found that circSATB2 was highly expressed in exosomes from NSCLC cells, especially in H1299 cells, compared with BEAS-2B cells (Fig. 5d); however, circSATB2 in exosomes removed from the culture medium was only weakly detected by qPCR. We therefore used H1299 cells for exosome preparation. circSATB2 level in circSATB2-sh and circSATB2-OE exosomes was also detected (Additional file 1: Figure S2A). To confirm exosomes were taken up by receptor cells, we stained nucleic acids in BEAS-2B, H460 and A549 cells with green fluorescence, then extracted the exosomes; the isolated exosomes were co-cultured with BEAS-2B, H460 and A549 cells, and fluorescent-labeled exosomes were observed in the receptor cells (Fig. 5e). We also transfected red fluorescent-labeled RNA sequences into exosomes as an extracellular stain, and fluorescence was observed in the receptor cells after 6 h incubation using a fluorescence microscope (Additional file 1: Figure S2B). These results indicated that exosomes were taken up by receptor cells. circSATB2 expression levels in BEAS-2B, H460 and A549 cells after co-culture with circSATB2-OE or circSATB2-sh exosomes were detected by qPCR (Fig. 5f). The results showed that expression of circSATB2 increased inBEAS-2B, H460 and A549 cells that were co-cultured with exosomes highly expressing circSATB2. We further investigated the roles of circSATB2 in cell proliferation, migration, and invasion via exosomes after co-culture with circSATB2-OE and circSATB2-sh exosomes in H460 and A549 cells. circSATB2-OE exosomes increased the proliferation compared with the lv5 group, while circSATB2-sh exosomes showed reduced capacity to affect cell proliferation in H460 and A549 cells compared with the lv3 group, as shown by EdU assay (Fig. 5g, h). circSATB2-OE exosomes increased the migration and invasion of H460 and A549 cells compared with lv5 group, as shown by wound healing assay (Fig. 5i, k) and Transwell migration assay (Fig. 5j, l), while circSATB2-sh exosomes produced lower efficiency on migration compared with lv3 group by Transwell invasion assay (Fig. 5m, n). Expression of miR-326 decreased,while *FSCN1* levels increased after co-culture with circSATB2 highly expressing exosomes that were increased in H460 and A549 cells (Additional file 1: Figure S2C, D). Furthermore, we found that exosomes with higher circSATB2 level could induce abnormal proliferation of BEAS-2B cells via CCK-8 cell viability assay (Fig. 5o) and colon formation assay (Fig. 5p, q). Overall, these results indicated that exosomes which contained circSATB2 can be taken up by BEAS-2B, H460 and A549 cells to participate in cell-cell communication, further affect the progression of NSCLC cells and proliferation of normal bronchial epithelial cells.

### Exosomal circSATB2 may act as a potential serum biomarker of NSCLC

**Fig. 6:**
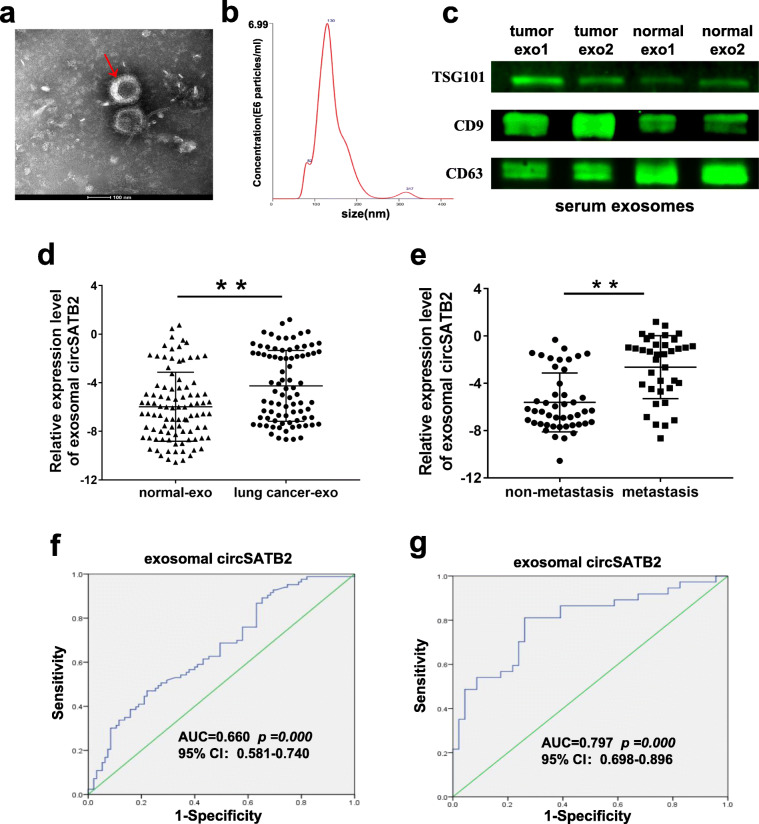
Exosomal circSATB2 expression in serum from NSCLC patients. **a** Identification of serum exosomes by TEM. **b** Size distribution of serumal exosome diameters. **c** Detection of TSG101, CD9 and CD63 protein expression by western blot. **d** circSATB2 expression in exosomes in serum from lung cancer patients and from non-cancerous donors detected by qPCR. **e** circSATB2 expression in exosomes from serum of patients with metastatic and non-metastatic lung cancer detected by qPCR. **f** ROC curve analyses and AUC values for circSATB2 in exosomes from serum of lung cancer patients and non-cancerous donors. **g** ROC curve analyses and AUC values for circSATB2 in exosomes from serum of lung cancer patients with metastatic and non-metastatic lung cancer. Experimental data presented as means±standard deviation. ^**^P < 0.01 compared with exosomes from serum of non-cancerous donors or patients with non-metastatic lung cancer

We previously showed that functional circSATB2 was highly expressed in lung cancer cell exosomes. We therefore examined the differential expression of circSATB2 in human serum exosomes. Exosomes were isolated from the serum of 83 NSCLC patients with lung cancer and 95 non-cancerous donors and identified (Fig. 6a-c). Nanoparticle tracker analysis (NTA) also showed a concentration of 3.68 × 10^9^ ± 3.61 × 10^8^/mL and zeta potential values of − 16.33 ± 3.019 mV, while the size distribution of the exosomes was within 200 nm, with a mode of 130.3 nm. circSATB2 was highly expressed in exosomes from serum of lung cancer patients compared with non-cancerous donors, as detected by qPCR (Fig. 6d). circSATB2 expression was also higher in exosomes from serum derived from patients with metastatic compared with non-metastatic lung cancer (Fig. 6e), suggesting that exosomal circSATB2 expression was related to lung cancer lymphatic metastasis. ROC curve analysis of exosomal circSATB2 showed an AUC value of 0.660 in serum from lung cancer patients (Fig. 6f) and an AUC value of 0.797 in serum from metastatic lung cancer patients (Fig. 6g). These results indicate that exosomal circSATB2 has the potential to act as a blood detection index for the diagnosis of lung cancer and lung cancer metastasis with high sensitivity and specificity.

## Discussion

Lung cancer has become the leading cause of cancer-related deaths worldwide with a rising trend of incidence and mortality, and NSCLC accounts for 85% of all lung cancer [3]. Most lung cancer cases are diagnosed at a middle or advanced stage when the treatment outcomes and prognosis are generally poor. ncRNAs have been shown to influence the occurrence and progression of cancer [26–28]. Non-coding circRNAs are widely expressed in human cells, and their expression levels may be much higher than those of the linear parental gene [29]. Compared with other ncRNAs, circRNAs are expressed more stably and specifically in cells, tissues and or body fluids [14, 30, 31] and thus have greater potential to act as effective biomarkers. We also previously revealed that circRNA circ NOL10 could function as a tumor suppressor in lung cancer [32]. circSATB2 (hsa_circ_0008928 in circBase) is an unreported circular RNA of 1004 nt. Previous studies on SATB2 showed its roles in disease, and SATB2 gene sets were associated with neurodevelopment [33–35], while the circSATB2 isoform lacked the capacity to encode protein. In the current study, we demonstrated that circSATB2 expression levels were significantly upregulated in NSCLC cells compared with normal bronchial epithelial cells. We then investigated the function of circSATB2 in NSCLC and found that circSATB2 knockdown inhibited the proliferation, migration, and invasion of NSCLC cells, while circSATB2 overexpression had the opposite effects, indicating that circSATB2 may promote the proliferation,migration and invasion of NSCLC cells.

Exosomes are secreted by all cell types and are naturally present in body fluids. Exosomes may also play important roles in the occurrence and development of tumors [36]. As an important biological carrier, exosomes participate in cell communication by transferring ncRNAs, with further potential effects on cancer progression. Exosomal miR-1247-3p induced cancer-associated fibroblast activation to foster lung metastasis of liver cancer [37], while exosome-transmitted lncARSR promoted sunitinib resistance in renal cancer [5], and exosomal circRNAs were found to promote the progression of hepatocellular carcinoma [38]. Circular RNAs in plasma exosomes of lung cancer have also been reported [39]. However, studies of exosomal circRNAs in NSCLC have not been performed to our knowledge. Our study therefore assessed circSATB2 expression in exosomes in the cell culture supernatant from normal and NSCLC cells and revealed high circSATB2 expression in NSCLC cell-derived exosomes. We barely detected circSATB2 expression when the exosomes were removed from culture medium, suggesting that circSATB2 mainly exists in exosomes. We further explored if circSATB2 also functioned by exosome transmission by extracting exosomes from circSATB2-sh and circSATB2-OE cells and co-culturing them with H460, A549 and BEAS-2B cells. Incubation with highly expressed exosomes increased the proliferation, migration, and invasion of H460 and A549 cells. Additionally, circSATB2 highly expressed exosomes could induce abnormal proliferation of BEAS-2B cells. These results suggested that circSATB2 could promote the proliferation, migration and invasion of NSCLC cells and increase the proliferation of normal bronchial epithelial cells via exosomes transfer. The molecular mechanisms of circRNAs in tumors are diverse. Circular RNAs have been reported to act as competing endogenous RNAs influencing the expression of miRNA target genes, thereby exerting regulatory functions at the post-transcriptional level [40, 41]. Our previous study found circRNA 100146 negatively regulate its activity in NSCLC by binding miR-361–3p and miR-615-5p [42]. We determined the subcellular localization of circSATB2 by FISH and by cytoplasm and nuclear localization and found that circSATB2 was mainly expressed in the cytoplasm. This suggested that circSATB2 may affect the function of lung cancer cells at the post-transcriptional level. Furthermore, bioinformatics analysis showed multiple miRNA-binding sites in the circSATB2 sequence, suggesting that circSATB2 might function via binding to miRNAs. We therefore predicted the miRNAs that might bind to circSATB2 using RegRNA and CSCD, chose miR31-5p, miR-326, and miR328-3p for further study. We determined if these miRNAs could bind to circSATB2 by dual luciferase reporter assay and showed that circSATB2 could directly bind to miR-326. We then detected miR-326 expression in circSATB2-sh and circSATB2-OE lung cancer cells and found that miR-326 expression was negatively regulated by circSATB2. As a tumor suppressor, miR-326 has been implicated in the proliferation and invasion of NSCLC cells [21]. miR-326 was also associated with poor prognosis and the promotion of growth and metastasis in gastric cancer [43], and inhibited epithelial-mesenchymal transition-induced cell invasion in lung adenocarcinoma [44]. Above studies have also shown that miR-326 participates in the development of lung cancer and other cancers. Overall, we concluded that circSATB2 could bind directly to miR-326 and negatively regulate miR-326 expression, thereby affecting the development of lung cancer.

We further studied the function of miR-326 in lung cancer by predicting the target mRNAs of miR-326 via miRBase and TargetScan. *FSCN1*, which has been closely associated with lung and other cancers, was chosen for further study. Knockdown of *FSCN1* suppressed the migration and invasion of NSCLC cells [45]. In addition, *FSCN1* was targeted by miR-200b and promoted the migration and invasion of NSCLC cells [46]. We verified the interaction between miR-326 and *FSCN1* by dual luciferase reporter assay and qPCR and showed that miR-326 could directly bind to the *FSCN1* 3′UTR and negatively regulate *FSCN1* expression. *FSCN1* was reported to bind miR-326 in gastric cancer [43], and our study supports the interaction between these two genes. We confirmed the ability of circSATB2 to regulate *FSCN1* via miR-326 by detecting *FSCN1* expression in circSATB2 knockdown or overexpressing NSCLC cells via qPCR and found that *FSCN1* expression was positively regulated by circSATB2. *FSCN1* expression also increased by circSATB2 highly expressing exosomes. Expression of *FSCN1* and circSATB2 were correlated in NSCLC cells. In addition, transfection with miR-326 mimics could rescue the upregulation of *FSCN1* levels induced by circSATB2 overexpression. FSCN1 protein expression was also positively regulated by circSATB2 and negatively regulated by miR-326. These results indicate that circSATB2 can regulate FSCN1 expression via binding to miR-326, thus further promoting the proliferation, migration and invasion of NSCLC cells.

We also detected the expression of circSATB2, miR-326 and *FSCN1* in NSCLC and normal adjacent tissues by qPCR. circSATB2 and *FSCN1* were upregulated while miR-326 was downregulated in lung cancer compared with normal adjacent tissues, as well as in metastatic compared with non-metastatic lung cancer tissues. Furthermore, analysis of the clinical characteristics of these lung cancer cohorts suggested that circSATB2 and *FSCN1* were positively related to lung cancer lymphatic metastasis, while miR-326 was negatively related. There was also a positive correlation between circSATB2 and *FSCN1* expression, while miR-326 was negatively correlated with circSATB2 and *FSCN1* expression. These results further demonstrate circSATB2 is related to NSCLC progression.

The early stage of lung cancer is often undiagnosed, resulting in delayed prognosis and poor treatment options. A blood biomarker would be helpful for convenient clinical diagnosis of NSCLC. Circular RNAs are abundant and more stable in exosomes [47], indicating the potential ability of exosomal circRNAs to serve as a biomarker in NSCLC. We found circSATB2 expression was higher in exosomes from serum of NSCLC patients compared with exosomes from non-cancerous donors, and was related to lung cancer lymphatic metastasis. ROC curve analysis demonstrated higher sensitivity and specificity for exosomal circSATB2 detection in exosomes from serum of NSCLC patients than from non-cancerous donors. These data support the potential of circSATB2 as an exosomal biomarker in lung cancer and metastatic lung cancer.

In summary, we demonstrated that circSATB2 plays a notable role in the progression of NSCLC. In NSCLC cells, circSATB2 may regulate FSCN1 expression via direct binding to miR-326 to further promote the cell proliferation, migration and invasion. Moreover, circSATB2 could participate in cell-to-cell communication and affect function of recipient cell. This study has provided the new evidence to our knowledge for the function of circSATB2, demonstrating a key role and the molecular mechanism of circRNAs in the development of NSCLC, and has revealed their potential as biomarkers for clinical detection. This study has thus provided a new perspective in research on circRNAs in NSCLC, as well as a scientific basis for novel developments in the diagnosis and treatment of lung cancer.

## Conclusions

Circular RNA circSATB2 can regulate FSCN1 expression via direct binding to miR-326 to further promote the progression of NSCLC, and may participate in cell-to-cell communication via exosomes. Exosomal circSATB2 may be a potential biomarker for clinical detection of NSCLC and metastatic NSCLC.

## Supplementary information

**Additional file 1: Figure S1.** The circSATB2 promoted proliferation, migration, and invasion of NSCLC cells. **Figure S2.** Identification and expression detection after exosomes treatment.

**Additional file 2: Table S1.** Primer sequences for quantitative real-time PCR. **Table S2.** Sequences of FISH probes. **Table S3.** Correlation between clinicopathological characteristics and expression of circSATB2 in 59 lung cancer and matched normal adjacent tissue. **Table S4.** Correlation between clinicopathological characteristics and expression of miR-326 in lung cancer and matched normal adjacent tissue. **Table S5.** Correlation between clinicopathological characteristics and expression of *FSCN1* in lung cancer and matched normal adjacent tissue.

## Data Availability

All data generated or analyzed during this study are included in this published article and its additional information files.

## Abbreviations

circRNA: Circular RNA
PCR: Polymerase Chain Reaction
EdU: 5-Ethynyl-2′-deoxyuridine
RNase R: Ribonuclease R
GAPDH: Glyceraldehyde-3-Phosphate Dehydrogenase
FISH: Fluorescence in situ hybridization
FSCN1: Fascin homolog 1, actin-bundling protein 1
TEM: Transmission electron microscopy
GAB1: GRB2-associated binding protein 1
PBS: Phosphate-buffered saline
