# Supplementary material for: Circular RNA circSATB2 promotes progression of non-small cell lung cancer cells
Source: Mol Cancer. 2020 Jun 3;19:101. doi: 10.1186/s12943-020-01221-6 (PMC7268724; doi:10.1186/s12943-020-01221-6)
Supplement: Supplementary file 1 — Additional file 1: Figure S1. The circSATB2 promoted proliferation, migration, and invasion of NSCLC cells. Figure S2. Identification and expression detection after exosomes treatment. [file 12943_2020_1221_MOESM1_ESM.doc]

**Supplementary Figures and Legends**

**Figure S1**


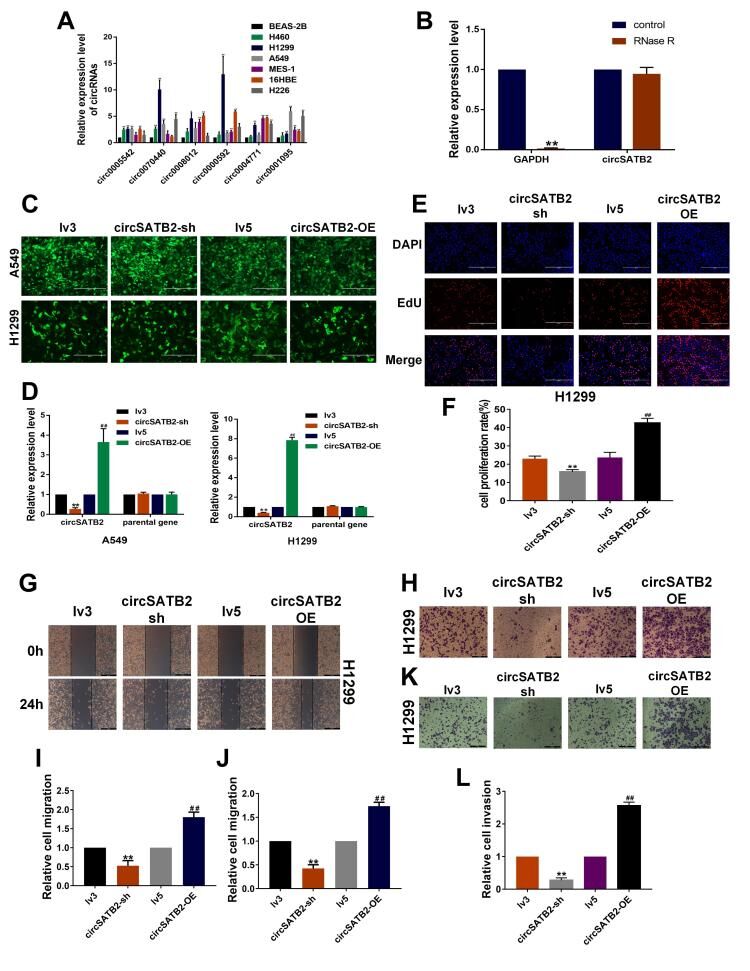


**Fig. S1. The circSATB2 promoted proliferation, migration, and invasion of NSCLC cells.** (A) The circRNAs expression in BEAS-2B and NSCLC cells. (B) Expression levels of circSATB2 after RNase R treatment detected by qRT-PCR. (C, D) Efficiency of stably transfected circSATB2 knockdown and overexpressing A549 and H1299 cells detected by [fluorescence](../../../../E:/Youdao/Dict/7.2.0.0703/resultui/dict/%3Fkeyword=fluorescence) [microscope](../../../../E:/Youdao/Dict/7.2.0.0703/resultui/dict/%3Fkeyword=microscope) (upper) and qPCR (under). (E, F) EdU assay to detect proliferation in circSATB2 knockdown and overexpressing stably transfected H1299 cells. (G, I) Wound healing assay to detect migration of circSATB2 knockdown and overexpression stably transfected H1299 cells. (H, J) Transwell migration assay to detect invasion of circSATB2 knockdown and overexpression stably transfected H1299 cells. (K, L) Transwell invasion assay to detect invasion of circSATB2 knockdown and overexpression stably transfected H1299 cells.

All experiments were repeated three times independently. Data are presented as means±standard deviation. **P*<0.05, ***P*<0.01 compared with BAES-2B cells or lv3 group. ##*P*<0.01 compared with lv5 group.

**Figure S2**


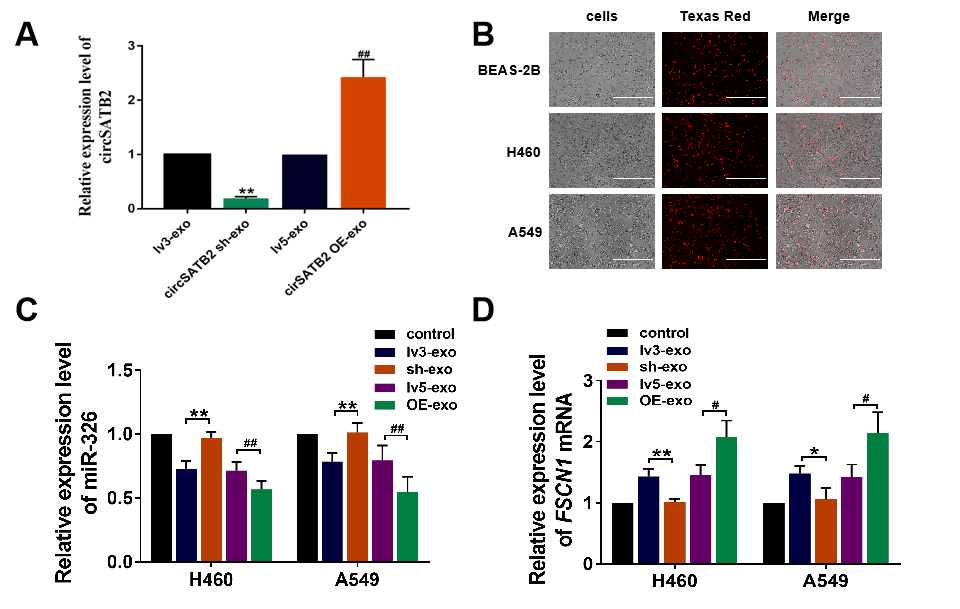


**Fig. S2.** **Identification and expression detection after exosomes treatment.** (A) circSATB2 expression in circSATB2-sh and circSATB2-OE exosomes of H1299 cells. (B) Exosomes transfected with fluorescence-labeled RNA sequence were taken up by recipient cells. (C) miR-326 expression after co-culture with circSATB2-sh and circSATB2-OE exosomes detected by qPCR. (D) FSCN1 mRNA expression after co-culture with circSATB2-sh and circSATB2-OE exosomes detected by qPCR.

All experiments were repeated three times independently. Data are presented as means±standard deviation. **P*<0.05, ***P*<0.01 compared with lv3-exo group; #*P*<0.05, ##*P*<0.01 compared with lv5-exo group.
