# Supplementary material for: Circular RNA circSATB2 promotes progression of non-small cell lung cancer cells
Source: Mol Cancer. 2020 Jun 3;19:101. doi: 10.1186/s12943-020-01221-6 (PMC7268724; doi:10.1186/s12943-020-01221-6)
Supplement: Supplementary file 2 — Additional file 2: Table S1. Primer sequences for quantitative real-time PCR. Table S2. Sequences of FISH probes. Table S3. Correlation between clinicopathological characteristics and expression of circSATB2 in 59 lung cancer and matched normal adjacent tissue. Table S4. Correlation between clinicopathological characteristics and expression of miR-326 in lung cancer and matched normal adjacent tissue. Table S5. Correlation between clinicopathological characteristics and expression of FSCN1 in lung cancer and matched normal adjacent tissue. [file 12943_2020_1221_MOESM2_ESM.doc]

**Supplementary Tables**

**Table S1. Primer sequences for quantitative real-time PCR**

| **Gene** | **Forward primer** | **Reverse primer** |
| --- | --- | --- |
| hsa_circ_0005542 | TTGTGGACAAGGCTTCCGAG | TGCAAAGCTACGTGGTCTCC |
| hsa_circ_0070440 | AAGGACGGTGATGTCTGCTC | AATGCCATTCTCGGTGAGCC |
| hsa_circ_0008012 | ACTACCTGGTGCCTCTAGTGA | GGCAGTGCCATCTGTGGTTG |
| hsa_circ_0000592 | TAGTCTCCAGGCATCAACCAC | TTCTGGGCACTCACCAAGTT |
| hsa_circ_0001095 | ATGGGTGGGCATGAACTCAG | TGATTCCCACTGCTGTCGAG |
| hsa_circ_0004771 | TCCGGATGACATCAGAGCTAC | CAAGTGTGCATCTTCTGGCTG |
| circSATB2 | AGCCAACCAACTCTTCCGTG | AAAGCACATCTTTCCGCACC |
| circSATB2-convergent | GCCCTGGTCTTCTTTCTCCC | CGGAAGAGTTGGTTGGCTCT |
| SATB2 | CAAGAGTGGCATTCAACCGCAC | ATCTCGCTCCACTTCTGGCAGA |
| cel-miR-39-3p | ACACTCCAGCTGGGTCACCGGGTGTAAATC | TGGTGTCGTGGAGTCG |
| GAPDH | GTCTCCTCTGACTTCAACAGCG | ACCACCCTGTTGCTGTAGCCAA |
| U6 | CTCGCTTCGGCAGCACA | AACGCTTCACGAATTTGCGT |
| FSCN1 | GACACCAAAAAGTGTGCCTTCCG | CAAACTTGCCATTGGACGCCCT |
| GAB1 | GGAAACTCTTGGCATTCAGGAGG | GCAGTCTGTTTCAGAAGAGGTGG |
| miR-326 | CGCCTCTGGGCCCTTC | AGTGCAGGGTCCGAGGTATT |
| miR-31 | GCGAGGCAAGATGCTGGC | AGTGCAGGGTCCGAGGTATT |
| miR-328 | GCTGGCCCTCTCTGCCC | AGTGCAGGGTCCGAGGTATT |

**Table S2. Sequences of FISH probes**

| **Gene symbol** | **Label** | **Probe sequence** |
| --- | --- | --- |
| circSATB2 | 6-FAM | GAATCATCAAACCTGTGTGCGGTTGAATGCC |

**Table S3. Correlation between clinicopathological characteristics and expression of circSATB2 in 59 lung cancer and matched normal adjacent tissue**

| **Characteristic** | **cases** | **Relative expression** | | ***p* value** |
| --- | --- | --- | --- | --- |
|  |  | **high** | **low** |  |
| Gender | | | | |
| male | 44 | 34 | 10 | 0.415 |
| female | 15 | 10 | 5 |  |
| Ages | | | | |
| ≤65 | 37 | 29 | 8 | 0.384 |
| >65 | 22 | 15 | 7 |  |
| Differentiation | | | | |
| well | 28 | 21 | 7 | 0.052 |
| moderate | 22 | 19 | 3 |  |
| poor | 9 | 4 | 5 |  |
| History type | | | | |
| adenocarcinoma | 38 | 28 | 10 | 0.800 |
| squamous carcinoma | 15 | 12 | 3 |  |
| others | 6 | 4 | 2 |  |
| Lymphatic metastasis | | | | |
| positive | 31 | 27 | 4 | 0.043^*^ |
| negative | 28 | 17 | 11 |  |
| Distant metastasis | | | | |
| yes | 9 | 7 | 2 | 1.000 |
| no | 50 | 37 | 13 |  |
| Primary location | | | | |
| left lung | 26 | 19 | 7 | 0.814 |
| right lung | 33 | 25 | 8 |  |
| TNM stage | | | | |
| Ⅰ-Ⅱ | 29 | 23 | 6 | 0.602 |
| Ⅲ-Ⅳ | 30 | 21 | 9 |  |
| Smoking history | | | | |
| smokers | 27 | 21 | 6 | 0.827 |
| never smokers | 32 | 23 | 9 |  |

** p<0.05*

**Table S4. Correlation between clinicopathological characteristics and expression of miR-326 in lung cancer and matched normal adjacent tissue**

| **Characteristic** | **cases** | **Relative expression** | | ***p* value** |
| --- | --- | --- | --- | --- |
|  |  | **high** | **low** |  |
| Sex | | | | |
| male | 44 | 15 | 29 | 0.833 |
| female | 15 | 4 | 11 |  |
| Ages | | | | |
| ≤65 | 37 | 9 | 28 | 0.093 |
| >65 | 22 | 10 | 12 |  |
| Differentiation | | | | |
| well | 28 | 10 | 18 | 0.112 |
| moderate | 22 | 4 | 18 |  |
| poor | 9 | 5 | 4 |  |
| History type | | | | |
| adenocarcinoma | 38 | 10 | 28 | 0.360 |
| squamous carcinoma | 15 | 7 | 8 |  |
| others | 6 | 2 | 4 |  |
| Lymphatic metastasis | | | | |
| positive | 31 | 7 | 24 | 0.029^*^ |
| negative | 28 | 13 | 15 |  |
| Distant metastasis | | | | |
| yes | 9 | 3 | 6 | 0.937 |
| no | 50 | 16 | 34 |  |
| Primary location | | | | |
| left lung | 26 | 8 | 18 | 1.000 |
| right lung | 33 | 11 | 22 |  |
| TNM stage | | | | |
| Ⅰ-Ⅱ | 29 | 9 | 20 | 1.000 |
| Ⅲ-Ⅳ | 30 | 10 | 20 |  |
| Smoking history | | | | |
| smokers | 27 | 9 | 18 | 0.865 |
| never smokers | 32 | 10 | 22 |  |

** p<0.05*

**Table S5. Correlation between clinicopathological characteristics and expression of *FSCN1* in lung cancer and matched normal adjacent tissue**

| **Characteristic** | **cases** | **Relative expression** | | ***p* value** |
| --- | --- | --- | --- | --- |
|  |  | **high** | **low** |  |
| Sex | | | | |
| male | 44 | 29 | 15 | 0.485 |
| female | 15 | 12 | 3 |  |
| Ages | | | | |
| ≤65 | 37 | 30 | 7 | 0.137 |
| >65 | 22 | 14 | 8 |  |
| Differentiation | | | | |
| well | 28 | 19 | 9 | 0.915 |
| moderate | 22 | 16 | 6 |  |
| poor | 9 | 6 | 3 |  |
| History type | | | | |
| adenocarcinoma | 38 | 25 | 13 | 0.620 |
| squamous carcinoma | 15 | 11 | 4 |  |
| others | 6 | 5 | 1 |  |
| Lymphatic metastasis | | | | |
| positive | 31 | 26 | 5 | 0.025^*^ |
| negative | 28 | 15 | 13 |  |
| Distant metastasis | | | | |
| yes | 9 | 8 | 1 | 0.327 |
| no | 50 | 33 | 17 |  |
| Primary location | | | | |
| left lung | 26 | 20 | 6 | 0.415 |
| right lung | 33 | 21 | 12 |  |
| TNM stage | | | | |
| Ⅰ-Ⅱ | 29 | 20 | 9 | 0.931 |
| Ⅲ-Ⅳ | 30 | 21 | 9 |  |
| Smoking history | | | | |
| smokers | 27 | 20 | 7 | 0.676 |
| never smokers | 32 | 21 | 11 |  |

** p<0.05*
